# Protonated Melamine Sponge for Effective Oil/Water Separation

**DOI:** 10.1038/srep14294

**Published:** 2015-09-24

**Authors:** Chih-Feng Wang, Hsiang-Ching Huang, Liang-Ting Chen

**Affiliations:** 1 Department of Materials Science and Engineering, I-Shou University, Kaohsiung, 840, Taiwan

## Abstract

In this study, we fabricated a superhydrophilic and underwater superoleophobic protonated melamine sponge for effective separation of water-rich immiscible oil/water mixtures with extremely high separation efficiency. This protonated melamine sponge exhibited excellent antifouling properties and could be used to separate oil/water mixtures continuously for up to 12 h without any increase in the oil content in filtrate. Moreover, our compressed protonated melamine sponge could separate both surfactant-free and -stabilized oil-in-water emulsions with high separation efficiencies. The high performance of this protonated melamine sponge and its efficient, energy- and cost-effective preparation suggest that it has great potential for use in practical applications.

Effective oil/water separation is a challenge worldwide because of the expanding production of industrial oily wastewater and the frequent oil spills arising from industrial accidents and the sinking of oil tankers and other ships. In 2010, the explosion of BP’s *Deepwater Horizon* oilrig resulted in 210 million gallons of oil being released into the Gulf of Mexico. Traditional techniques for oil-water separation (e.g., gravity separation combined with skimming, flotation, oil-adsorbing materials, flocculation, and coagulation)^1^,^2^ have low separation efficiencies and high energy costs, and require complex separation steps. As a result, there is a need to develop new materials that would allow oil/water separations to be performed efficiently, at low cost, with high selectivity^3^,^4^,^5^. Recently, materials possessing both superhydrophobic and superoleophilic properties have attracted broad attention because of their capacity to mediate the efficient separation of oils, organic pollutants, and other hydrophobic organic solvents from water^6^,^7^,^8^,^9^,^10^,^11^,^12^. Although such previously developed materials can be effective agents for oil/water separation, they are readily fouled, or even blocked up, by oils because of their intrinsic oleophilicity. Furthermore, because water is generally denser than oil, it tends to settle below an oil phase, forming a barrier layer above the separation material and inhibiting oil permeation into it. Accordingly, materials possessing superhydrophobicity and superoleophilicity are not suitable for the separation of water-rich oil/water mixtures or oil-in-water emulsions.

Inspired by the wetting behavior of fish scales, and taking advantage of high-energy materials having water-favoring properties, it has been possible to construct underwater superoleophobic surfaces in oil/water/solid three-phase systems^13^. Following this strategy, several materials possessing both superhydrophilicity and underwater superoleophobicity have been prepared, using various methods, with the aim of using them for efficient oil/water separations^14^,^15^,^16^,^17^,^18^,^19^,^20^,^21^,^22^,^23^. Feng *et al.* described a superhydrophilic and underwater superoleophobic hydrogel-coated mesh for the efficient separation of oil/water mixture^14^. Jin and coworkers developed nanowire-haired inorganic membranes possessing superhydrophilicity and underwater ultralow adhesive superoleophobicity that could effectively separate both immiscible oil/water mixtures and oil-in-water emulsions with high separation efficiencies^15^. They also used a sol-gel method to prepare superhydrophilic single-walled carbon nanotube/TiO_2_ nanocomposite films, which were applicable for the separation of both a surfactant-free emulsion and a surfactant-stabilized oil-in-water emulsion^16^. More recently, underwater superoleophobic graphene-based materials have been prepared and applied in the separation of oil/water mixtures^17^. With their high separation efficiencies, separation capacities, and anti-oil fouling properties, these materials have promising applications for treating wastewaters produced industrially, in daily life, and from oil spills.

In this paper, we present a simple and inexpensive dipping method for the fabrication of a superhydrophilic and underwater superoleophobic protonated melamine sponge. Melamine sponges are commercially available three-dimensional porous materials exhibiting superhydrophilicity and underwater superoleophobicity. Melamine sponges pre-wetted with water display under-oil superhydrophilicity and water adsorption ability and could be used in effective oil/water separation. In continuous oil/water separation experiments of diesel/water mixtures, however, the diesel would permeate through the melamine sponge. When protonated with HCl to enhance its oleophobicity, the as-prepared protonated melamine sponge exhibited high separation capacity, allowing separation of oil/water mixtures continuously for up to 12 h without any increase in the oil content in the filtrate. Interestingly, we found that the compressed protonated melamine sponge could separate both surfactant-free and -stabilized oil-in-water emulsions, solely driven by gravity, with high separation efficiencies. The excellent performance of the protonated melamine sponge in oil/water separations and its preparation through an industrially feasible process suggest that it has potential applicability in both academic and industrial settings.

## Results

**Figure 1:**
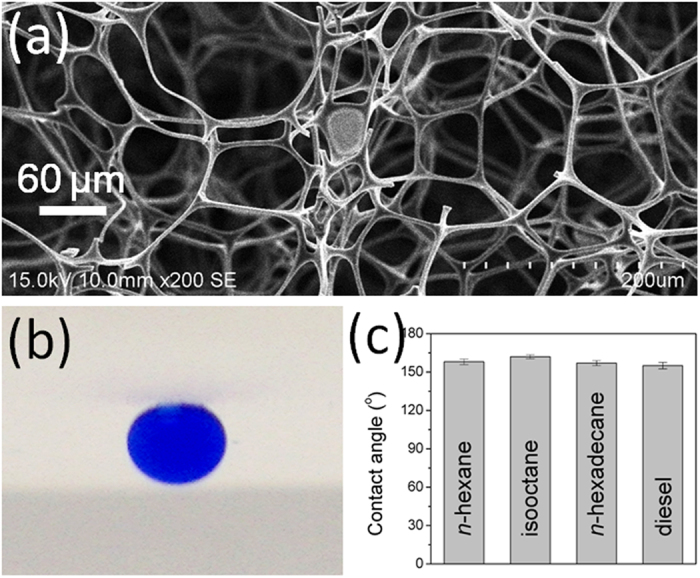
(**a**) SEM image of a pristin melamine sponge. (**b**) Photograph of an oil droplet (*n*-hexane) beneath a melamine sponge in water (contact angle: 158°). (**c**) Underwater contact angle of a series of oils.

Commercially available melamine sponges are effective abrasive cleaners because of their great hardness and open-cell porous microstructures (Fig. 1a). To investigate the wetting behavior of water and oil toward a melamine sponge, we used a charge-coupled device camera system to record the spreading of water and oil droplets. When a water droplet (4 μL) came into contact with the surface, it spread out and permeated into the melamine sponge instantly, resulting in a contact angle of approximately 0°. The same situation occurred when using a droplet of oil as a detecting probe. Both processes were complete within 1 s, suggesting both hydrophilicity and oleophilicity of the melamine sponge in air.

Owing to Young’s equation, the contact angle of oil in water on a flat surface can be expressed as the following^23^:


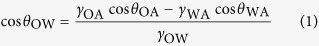


**Figure 2:**
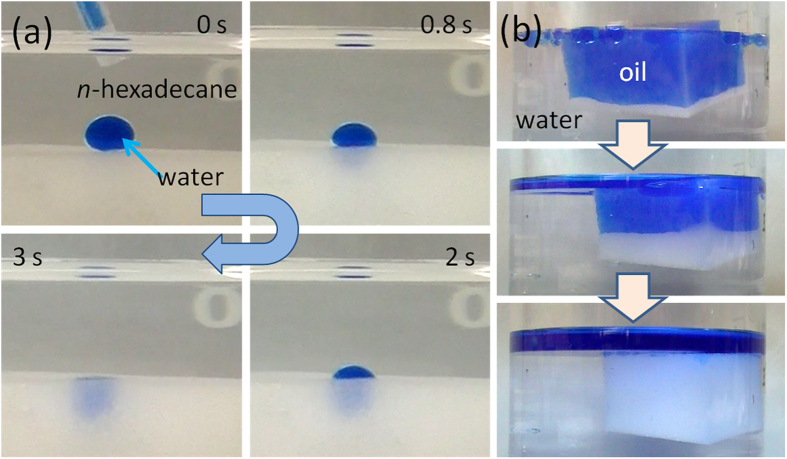
A series of photographs of (**a**) the spreading and permeating behavior of a water droplet on the melamine sponge under oil and (**b**) the self-cleaning anti-oil ability of the melamine sponge.

Where 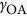 is the the oil/air interface tension, 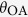 is the contact angle of oil in air, 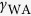 is the water/air interface tension, 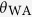 is the contact angle of water in air, 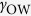 is the oil/water interface tension and is the contact angle of oil in water. Since the surface tension of oil and organic liquids is much lower than that of water, we can see that hydrophilic surfaces in air can become oleophobic in water. The melamine sponge became superoleophobic when immersed in water. Underwater oil droplets were nearly spherical on the melamine sponge surface (Fig. 1b) and exhibited high contact angles (>150°; Fig. 1c). The rough surface structure and superhydrophilicity of the melamine sponge combined to result in a particular wettability characterizing an oil/water/solid three-phase system. When we immersed the melamine sponge in water, water became trapped within its rough microstructure that then formed an oil/water/solid composite interface in the presence of oil^13^,^18^,^24^. The trapped water molecules significantly decreased the contact area between the oil and the surface of the melamine sponge, leading to underwater superoleophobicity. For an oil/water/solid system with a rough surface, the contact angle could be described with the help of the Cassie−Baxter equation^25^:


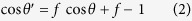


where f is the area fraction of the solid, 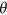
is the contact angle of the oil droplet on a smooth surface in water, 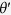
and is the contact angle of the oil droplet on a rough surface in water. A smaller area fraction indicates a lower opportunity of the oil droplet contacting the solid surface, and the larger the contact angle of oil in water. The melamine sponges possess a very rough surface, which intimates a rather small area fraction of solid and a large oil contact angle. The pre-wetted melamine sponge (in the pre-wetting process, the melamine sponge was immersed in water for 30 min and then squeezed to form a water layer on the skeleton of the melamine sponge) exhibited superhydrophilicity, even under oil. Figure 2a displays the under-oil (*n*-hexadecane) wettability of a pre-wetted melamine sponge: the water droplet (4 μL) was adsorbed once it made contact with the pre-wetted melamine sponge. The pre-wetted melamine sponge also exhibited under-oil superhydrophilicity and water adsorption ability in various other organic solvents and oils, including *n*-hexane, isooctane, and diesel. Figure 2b presents the results of a self-cleaning anti-oil test performed using a pre-wetted melamine sponge. The pre-wetted melamine sponge was filled with *n*-hexadecane (labeled with blue dye). When we immersed it below the surface of water, the water displaced the oil in a continuous manner. Eventually, the pre-wetted melamine sponge became saturated with water, without any absorbed *n*-hexadecane, revealing its self-cleaning anti-oil properties. Such self-cleaning anti-oil properties of pre-wetted melamine sponge may just happen on account of hydration of polymer surface. The thin layer of water formed on the polymer and not allows the oil to contact the surface.

**Figure 3:**
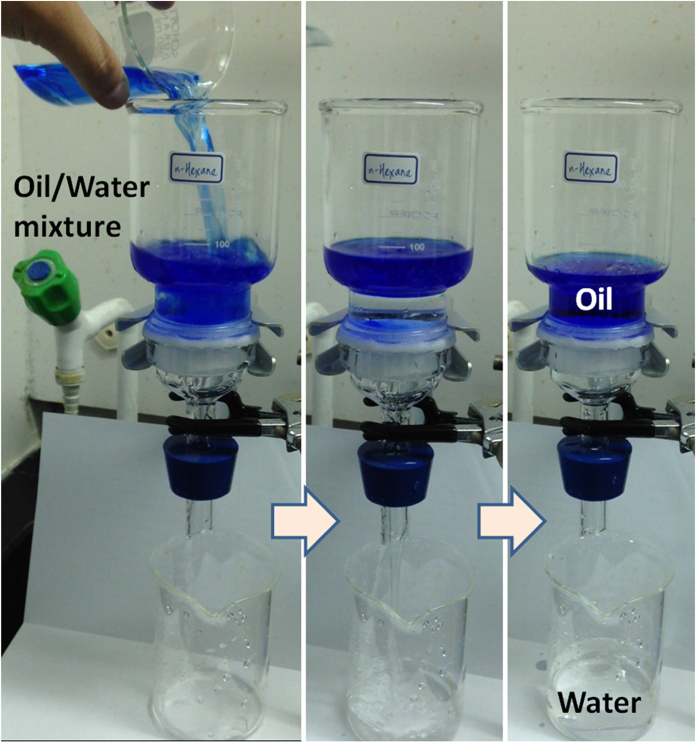
Solely gravity-driven separation for oil/water mixtures, performed using the melamine sponge.

As expected, the pre-wetted melamine sponge, which exhibited both underwater superoleophobicity and water-permeability, facilitated the efficient separation of oil/water mixtures. Figure 3 displays the procedure that we followed to perform the oil/water separation experiment. We fixed a melamine sponge between two glass tubes and then poured 150 mL of a mixture of *n*-hexane and water (1:2, v/v) into the upper tube. The water quickly passed through the pre-wetted melamine sponge and entered the beaker below. Meanwhile, all of the oil was retained above the sponge, due to the underwater superoleophobicity of the pre-wetted melamine sponge. No external force was applied during this rapid separation process (within 10 s), only gravity. Using this approach, we successfully separated several oil/water and organic solvent/water mixtures, including those containing *n*-hexadecane, isooctane, and diesel. To further investigate the robustness and antifouling properties of the melamine sponge, we performed a continuous oil/water separation test, adding water into the upper glass tube continuously while maintaining the height of the oil/water mixture at 6.5 cm. Several oil/water mixtures, including those containing *n*-hexane, *n*-hexadecane, and isooctane, could separated in these continuous separation tests over a period of at least 1 h. We used a gas chromatography with flame ionization detection (GC/FID) system to measure the oil contents of the filtrates. Table 1 reveals, for each of the tested immiscible oil/water mixtures, the oil content in the separated water was less than 2.0 ppm, confirming the high separation efficiency provided by the melamine sponge. We also calculated the fluxes of the melamine sponge by measuring the time required for complete permeation of a certain volume of the oil/water mixtures. Each of the oil/water mixtures exhibited a high flux during the continuous separation test; the *n*-hexane/water, isooctane/water, and *n*-hexadecane/water mixtures provided comparable fluxes of 95000, 79700, and 86700 L m^−2^ h^−1^ (Table 1).

**Figure 4:**
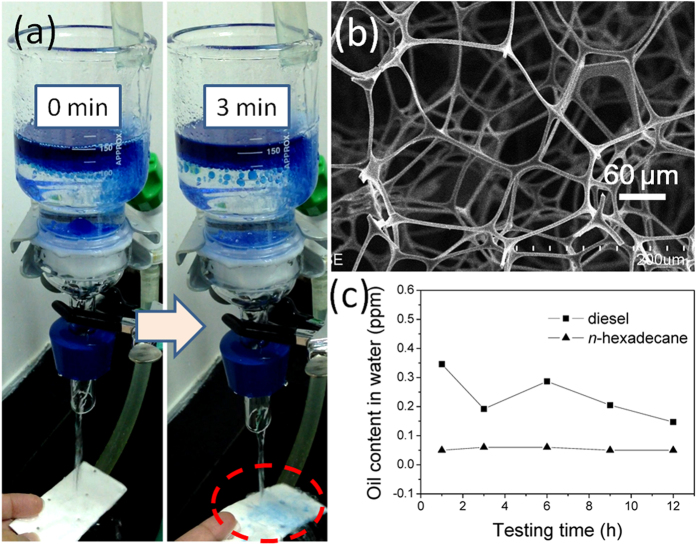
(**a**) Continuous separation test for diesel/water mixtures, performed using the melamine sponge. (**b**) SEM image of protonated melamine sponge. (**c**) Real-time monitoring of the variation in oil content in the filtrate, plotted with respect to time, during up to 12 h of separation of diesel/water and *n*-hexadecane/water mixtures, performed using protonated melamine sponges.

Nevertheless, in the continuous diesel/water separation test, we found that diesel passed through the melamine sponge within 3 min (Fig. 4a). Accordingly, we protonated the melamine sponge with HCl to enhance its oleophobicity and antifouling properties. Figure 4b presents SEM images of the protonated melamine sponge, which retained the microstructure of the pristine melamine sponge but exhibited superior antifouling properties. Using the protonated melamine sponge, we could perform continuous separations of diesel/water and n-hexadecane/water mixtures for up to 12 h, with real-time detection of the oil content in the filtrate. We did not detect any oil in the collected water during the entire testing process, indicating the effectiveness of the separation of the oil/water mixture using the protonated melamine sponge; the oil contents in both filtrates remained at less than 0.4 ppm (Fig. 4c). Thus, it should be possible to use the protonated melamine sponge to treat large amounts of oil/water mixtures over long periods of time because of its excellent antifouling properties.

**Figure 5:**
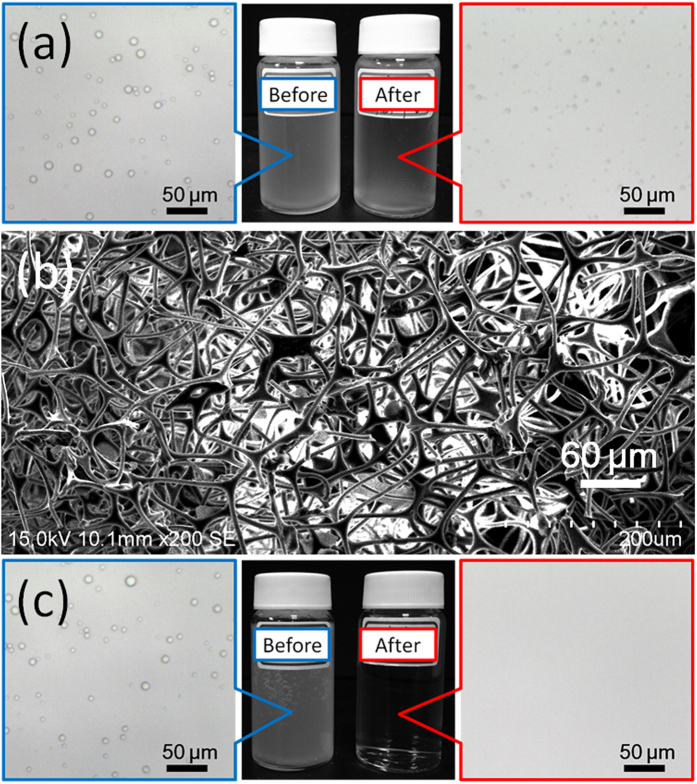
(**a**) Photographs of the surfactant-free *n*-hexane–in–water emulsion before and after separation performed using a protonated melamine sponge. (**b**) SEM image of a compressed protonated melamine sponge. (**c**) Photographs of the surfactant-free *n*-hexane–in–water emulsion before and after separation performed using a compressed protonated melamine sponge.

**Figure 6:**
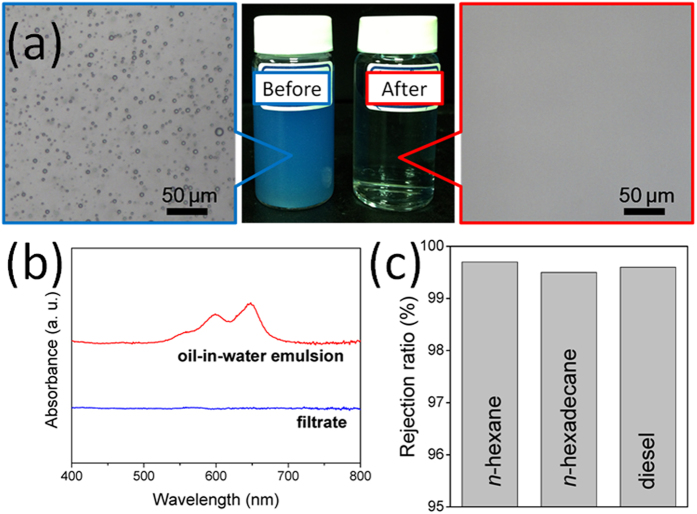
Using a compressed protonated melamine sponge for the demulsification and separation of a surfactant-stabilized oil-in-water emulsion. (**a**) Photographs and (**b**) UV−V is spectra of the surfactant-free *n*-hexane–in–water emulsion before and after separation. (**c**) Oil rejection ratios of three kinds of filtrates collected from their emulsions.

Emulsified oil in wastewater is also a major environmental issue affecting a range of industries. Direct discharge of such wastewater harms both the environment and human health. Therefore, developing efficient, energy- and cost-effective processes for the removal of emulsified oil from wastewater remains desirable, yet challenging. Because of its large pores (>50 μm, Fig. 4b), the protonated melamine sponge could separate only free oil/water mixtures; it could not separate oil-in-water emulsions, where the typical droplet size is less than 20 μm. Figure 5a displays optical microscopy images of a surfactant-free *n*-hexane–in–water emulsion and the corresponding collected filtrate; the presence of oil droplets indicates that the protonated melamine sponge was not suitable for the separation of this emulsified oil/water mixture. Interestingly, we found that the protonated melamine sponge could be used successfully for the separation of emulsified oil/water after applying a simple compression process. Figure 5b displays an SEM micrograph of the compressed protonated melamine sponge. Compared with the original film (Fig. 4b), the surface morphology changed such that the pores had smaller diameters and that the skeleton of the melamine sponge was packed more compactly. Figure 5c presents the results of separating the surfactant-free *n*-hexane–in–water emulsion through the compressed protonated melamine sponge. The collected filtrate was transparent, whereas the original feed emulsion was milky white. In addition, optical microscopy images revealed a significant difference in phase composition between the feed emulsion and the resulting filtrate. We did not observe any droplets in the collected filtrate, implying that the *n*-hexane had been removed from the surfactant-free *n*-hexane–in–water emulsion. The oil contents in the collected filtrates from the *n*-hexane, *n*-hexadecane, and diesel emulsions were 1.88, 0.28, and 2.87 ppm, respectively, suggesting exceedingly high separation efficiencies. The compressed protonated melamine sponge also possessed high efficiency when separating surfactant-stabilized oil-in-water emulsions. The water permeated through the sponge continuously, causing the emulsion droplets to demulsify, leaving behind the oil. Figure 6a reveals that the components of a surfactant-stabilized oil-in-water emulsion could be separated well and collected in a single step (here, the oil was dyed with Sudan Blue II). Similar to the results we obtained when separating surfactant-free oil-in-water emulsions, we observed no oil droplets in the image of the filtrate, confirming that the compressed protonated melamine sponge was effective for the separation of the surfactant-stabilized oil-in-water emulsion. We also used a UV–Vis spectrophotometer to analyze the purified water. The absence of any characteristic peaks for Sudan Blue II dissolved in oil in the spectrum (Fig. 6b) and the rejection ratio of oil of greater than 99.5% (Fig. 6c) illustrate the high separation efficiency of the compressed protonated melamine sponge.

## Discussion

We have developed a facile, inexpensive method for the fabrication of a protonated melamine sponge that displays superhydrophilicity and underwater superoleophobicity; this material should have practical application in the effective separation of water-rich immiscible oil/water mixtures with extremely high separation efficiency and separation capacity. The protonated melamine sponge exhibited excellent antifouling properties during long-term use. Moreover, after performing a simple compression process, the protonated melamine sponge allowed the effective separation of surfactant-free and -stabilized water-in-oil emulsions with high separation efficiency. We believe that this kind of sponge is a promising candidate material for use in the treatment of wastewater produced industrially and in daily life.

## Methods

### Materials

Commercial melamine sponges were purchased from BASF. Tween 80 was supplied by Acros. Sudan Blue II was purchased from Sigma Aldrich.

### Protonated Melamine Sponge

The melamine sponge was soaked in 1.0 M HCl for 30 min and then washed multiple times with DI water. In the compression procedure, the melamine sponge was compressed into a compact form having a volume that was approximately one fourteenth that of the pristine sample.

### Oil-in-Water Emulsions

To prepare a surfactant-free *n*-hexane–in–water emulsion, *n*-hexane (20 mL) was added into water (180 mL) and then the mixture was sonicated for 30 min. To prepare surfactant-free *n*-hexadecane-in-water and diesel-in-water emulsions, the oil and water (1:15, v:v) were mixed and then sonicated for 30 min to produce white solutions. To prepare surfactant-stabilized oil-in-water emulsions, Tween 80 (0.02 g) was dissolved in water (200 mL), an oil (*n*-hexane, *n*-hexadecane, or diesel; 2.0 mL) was added, and then the mixture was stirred for 1 h.

### Oil/Water Separation Experiment

The pristine and protonated melamine sponges were fixed between two glass vessels having a diameter of 34 mm. The compressed melamine sponge was fixed in a syringe having a diameter of 19 mm. All sponges used for oil/water separations were pre-wetted with water. The oil/water mixtures were poured into the filter and the separation was performed driven by gravity. The filtrate water was collected and its oil content determined using gas chromatography with flame ionization detection [GC/FID; 5890 (HP) and 7890 (Agilent)]. The oil rejection ratios in the filtrate from the surfactant-stabilized oil-in-water emulsions were determined by measuring the oil contents in the feed and corresponding filtrates, using a UV–Vis spectrophotometer (Evolution 201, Thermo Scientific; the oil was dyed with Sudan Blue II).

### Instruments and Characterization

The microstructures of the pristine, protonated, and compressed melamine sponges were characterized using a HITACHI S-3400 scanning electron microscope (acceleration voltage: 15.0 kV). Static contact angles of droplets (5 μL) were measured using an FDSA MagicDroplet-100 contact angle goniometer; each reported contact angle represents the average of six measurements. Optical microscopy images were recorded using an Olympus BX51M instrument after placing a drop of an emulsion solution onto a biological counting board.

## Additional Information

**How to cite this article**: Wang, C.-F. *et al.* Protonated Melamine Sponge for Effective Oil/Water Separation. *Sci. Rep.* **5**, 14294; doi: 10.1038/srep14294 (2015).

## Supplementary Material

Supporting Information

Supplementary Video

## Figures and Tables

**Table 1:** The oil content in filtrate and flux of the melamine sponges during continuous oil/water separation.

Mixture	Oil content infiltrate (ppm)	Flux (L m^−2^ h^−1^)
*n*-hexane/water	1.60	95000
isooctane/water	0.27	79700
*n*-hexadecane/water	0.05	86700

## References

[b1] Al-Shamrani A. A., James A. & Xiao H. Destabilisation of oil-water emulsions and separation by dissolved air flotation. Water Res. 36, 1503–1512 (2002). 11996340 10.1016/s0043-1354(01)00347-5

[b2] Rubio J., Souza M. L. & Smith R. W. Overview of flotation as a wastewater treatment technique. Miner. Eng. 15, 139–155 (2002).

[b3] Dalton T. & Jin D. Extent and frequency of vessel oil spills in US marine protected areas. Mar. Pollut. Bull. 60, 1939–1945 (2010). 20797735 10.1016/j.marpolbul.2010.07.036

[b4] Li H. L. & Boufadel M. C. Long-term persistence of oil from the Exxon Valdez spill in two-layer beaches. Nat. Geosci. 3, 96–99 (2010).

[b5] Yip T. L., Talley W. K. & Jin D. The effectiveness of double hulls in reducing vessel-accident oil spillage. Mar. Pollut. Bull. 62, 2427–2432 (2011). 21924747 10.1016/j.marpolbul.2011.08.026

[b6] Liu K. S., Tian Y. & Jiang L. Bio-inspired superoleophobic and smart materials: Design, fabrication, and application. Prog. Mater. Sci. 58, 503–564 (2013).

[b7] Pan Y. X. *et al.* Evaluation of Hydrophobic Polyvinyl-Alcohol Formaldehyde Sponges As Absorbents for Oil Spill. ACS Appl. Mater. Interfaces 6, 8651–8659 (2014). 24797603 10.1021/am5014634

[b8] Pham V. H. & Dickerson J. H. Superhydrophobic Silanized Melamine Sponges as High Efficiency Oil Absorbent Materials. ACS Appl. Mater. Interfaces 6, 14181–14188 (2014). 25039789 10.1021/am503503m

[b9] Ruan C. P., Ai K. L., Li X. B. & Lu L. H. A Superhydrophobic Sponge with Excellent Absorbency and Flame Retardancy. Angew. Chem.-Int. Edit. 53, 5556–5560 (2014). 10.1002/anie.201400775 24711147

[b10] Wang C. F. & Lin S. J. Robust Superhydrophobic/Superoleophilic Sponge for Effective Continuous Absorption and Expulsion of Oil Pollutants from Water. ACS Appl. Mater. Interfaces 5, 8861–8864 (2013). 24032484 10.1021/am403266v

[b11] Wang C. F., Tzeng F. S., Chen H. G. & Chang C. J. Ultraviolet-Durable Superhydrophobic Zinc Oxide-Coated Mesh Films for Surface and Underwater-Oil Capture and Transportation. Langmuir 28, 10015–10019 (2012). 22679902 10.1021/la301839a

[b12] Zhang W. B. *et al.* Superhydrophobic and Superoleophilic PVDF Membranes for Effective Separation of Water-in-Oil Emulsions with High Flux. Adv. Mater. 25, 2071–2076 (2013). 23418068 10.1002/adma.201204520

[b13] Liu M. J., Wang S. T., Wei Z. X., Song Y. L. & Jiang L. Bioinspired Design of a Superoleophobic and Low Adhesive Water/Solid Interface. Adv. Mater. 21, 665–669 (2009).

[b14] Xue Z. X. *et al.* A Novel Superhydrophilic and Underwater Superoleophobic Hydrogel-Coated Mesh for Oil/Water Separation. Adv. Mater. 23, 4270–4273 (2011). 22039595 10.1002/adma.201102616

[b15] Zhang F. *et al.* Nanowire-Haired Inorganic Membranes with Superhydrophilicity and Underwater Ultralow Adhesive Superoleophobicity for High-Efficiency Oil/Water Separation. Adv. Mater. 25, 4192–4198 (2013). 23788392 10.1002/adma.201301480

[b16] Gao S. J., Shi Z., Zhang W. B., Zhang F. & Lin J. Photoinduced Superwetting Single-Walled Carbon Nanotube/TiO2 Ultrathin Network Films for Ultrafast Separation of Oil-in-Water Emulsions. ACS Nano 8, 6344–6352 (2014). 24869793 10.1021/nn501851a

[b17] Dong Y. *et al.* Underwater superoleophobic graphene oxide coated meshes for the separation of oil and water. Chem. Commun. 50, 5586–5589 (2014). 10.1039/c4cc01408a 24722821

[b18] Tao M. M., Xue L. X., Liu F. & Jiang L. An Intelligent Superwetting PVDF Membrane Showing Switchable Transport Performance for Oil/Water Separation. Adv. Mater. 26, 2943–2948 (2014). 24677285 10.1002/adma.201305112

[b19] Chen P. C. & Xu Z. K. Mineral-Coated Polymer Membranes with Superhydrophilicity and Underwater Superoleophobicity for Effective Oil/Water Separation. Sci. Rep. 3, 6 (2013). 10.1038/srep02776 PMC3784956 24072204

[b20] Yoon H. *et al.* Gravity-Driven Hybrid Membrane for Oleophobic-Superhydrophilic Oil Water Separation and Water Purification by Graphene. Langmuir 30, 11761–11769 (2014). 25192514 10.1021/la5031526

[b21] Raza A. *et al.* *In situ* cross-linked superwetting nanofibrous membranes for ultrafast oil-water separation. J. Mater. Chem. A 2, 10137–10145 (2014).

[b22] Zhang W. B. *et al.* Salt-Induced Fabrication of Superhydrophilic and Underwater Superoleophobic PAA-g-PVDF Membranes for Effective Separation of Oil-in-Water Emulsions. Angew. Chem.-Int. Edit. 53, 856–860 (2014). 10.1002/anie.201308183 24307602

[b23] Xue Z. X., Cao Y. Z., Liu N., Feng L. & Jiang L. Special wettable materials for oil/water separation. J. Mater. Chem. A 2, 2445–2460 (2014).

[b24] Liu M. J., Zheng Y. M., Zhai J. & Jiang L. Bioinspired Super-antiwetting Interfaces with Special Liquid-Solid Adhesion. Accounts Chem. Res. 43, 368–377 (2010). 10.1021/ar900205g 19954162

[b25] Cassie A. B. D. & Baxter S. Wettability of Porus Surfaces. Trans. Faraday Soc. 40, 546−551 (1944).

