# Supplementary material for: Protonated Melamine Sponge for Effective Oil/Water Separation
Source: Sci Rep. 2015 Sep 24;5:14294. doi: 10.1038/srep14294 (PMC4585846; doi:10.1038/srep14294)
Supplement: Supporting Information [file srep14294-s1.doc]

**Supporting Information**

**Protonated Melamine Sponge for Effective Oil/Water Separation**

Chih-Feng Wang*, Hsiang-Ching Huang, Liang-Ting Chen

Department of Materials Science and Engineering, I-Shou University, Kaohsiung, 840, Taiwan.

*To whom all correspondence should be addressed

E-mail: [cfwang@isu.edu.tw](mailto:cfwang@isu.edu.tw)

Tel: 886-7-6577711-3129

Fax: 886-7-6578444

Video legend: Video clips of the oil/water separation process through the protonated melamine sponge.
